# Monothalamous soft-shelled foraminiferal image dataset from the Kveithola Trough (NW Barents Sea)

**DOI:** 10.1016/j.dib.2023.109603

**Published:** 2023-09-22

**Authors:** F. Caridi, A. Sabbatini, C. Morigi

**Affiliations:** a Marche Polytechnic University, Department of Life and Environmental Sciences (DiSVA), Via Brecce Bianche 12, 60131 Ancona, Italy; b Department of Earth Sciences, Università di Pisa, 56126 Pisa, Italy

**Keywords:** Monothalamous soft-shelled taxa, Benthic foraminifera, Arctic, Biodiversity

## Abstract

We present an image dataset of monothalamous soft-shelled Foraminifera (Monothalamea, [1]), an important component of benthic foraminiferal assemblage in sediment cores collected during two oceanographic expeditions that contributed to the MSM30-CORIBAR project (Ice dynamics and meltwater deposits: coring in the Kveithola trough, NW Barents Sea). 9 subsamples of sediment cores were collected during different years (2013–2016) in the Kveithola Trough, a glacially carved system in the NW Barents Sea. Cores were retrieved using a multi-corer (MUC) and a giant box-corer (GBC) and the subcores for foraminiferal analyses were obtained using Plexiglas tubes inserted manually into the cores. These subcores were sliced at 0.5 cm intervals down to 2 cm sediment depth and then every 1 cm down to 10 cm. Two staining methods, Cell Tracker Green (CTG) and Rose Bengal (RB), were used to distinguish between living and dead individuals. Then, the fixed sediment samples were sieved through 63 and 150 μm mesh screens and preserved in 10 % borax-buffered formalin. Six species and 37 undescribed morphotypes were recognized and included in this image dataset.

Relatively few species of soft-shelled, monothalamous foraminifera have been described compared to a much larger number of undescribed morphotypes recognised from across the marine realm. Few researchers study with their taxonomy because of the time and difficulties that morphological identification involves. In addition, because “soft”, delicate monothalamids rarely fossilize, they are generally overlooked by micropaleontologists. However, they are abundant and diverse and represent an important faunal component of marine as well as freshwater ecosystems. Further information about these frequently overlooked protists will help to address important knowledge gaps and enhance our ability to manage and conserve the planet's resources responsibly. In particular, our image dataset highlights the importance of monothalamous soft-shelled foraminifera in this peculiar Arctic environment and contributes to the first species/morphotype checklist for the area. We hope it will serve to fill gaps in knowledge regarding the ecology and biodiversity of benthic foraminifera, helping users to identify monothalamids species and morphotypes in Arctic waters and beyond.

This data article is associated with the research papers: “Benthic foraminiferal assemblages and environmental drivers along the Kveithola Trough (NW Barents Sea)” by [2].

Specifications Table

**Table:** 

Subject	Ecology
Specific subject area	Taxonomic work of soft-shelled monothalamous foraminiferal species/ morphotypes from the Arctic latitudes that provides new data about their abundance and biodiversity.
Data format	Analyzed
Type of data	Table, Image
Data collection	The data were acquired during two oceanographic expeditions in 2013 and 2016 in the Kveithola Trough (NW Barents Sea) as part of the MSM30-CORIBAR project: R/V Maria S. Merian, and R/V Polarstern cruise PS99–1a, Eurofleets2-BURSETER. Samples were obtained using Plexiglas cores inserted manually into multi-corer (MUC) or giant box-corer (GBC) samples. Two staining methods, Cell Tracker Green (CTG) and Rose Bengal (RB), were used to distinguish living from dead individuals. Samples were hand-sorted in water, using a simple stereomicroscope (Nikon SMZ 645) and fluorescence stereomicroscope (Leica M205 FCA) for RB and CTG-stained samples, respectively.
Data source location	Sediment cores were collected in the Kveithola Trough, a glacially carved system located in the NW Barents Sea. Site coordinates MSM30-CORIBAR cruise:
	• Site **01**, 74°51, 53′ N 16°05, 93′ E • Site **07,** 74°50, 74′ N 17°38, 35′ E • Site **20**, 74°50, 75′ N 18°10, 55′ E • Site **22**, 74°59, 69′ N 17°59, 72′ E Site coordinates Eurofleets2-BURSETER cruise:
	• Site **02**, 74°51, 53′ N 16°05, 93′ E • Site **21,** 74°52, 40′ N 17°21, 57′ E • Site **05,** 74°51, 53′ N 17°38, 37′ E • Site **06**, 74°50, 75′ N 18°10, 55′ E • Site **07,** 74°59, 69′ N 17°59, 72′ E
Data accessibility	Repository name: Mendeley Data identification number: 10.17632/zrynydx7py.1
Related research article	1. Caridi, F., Sabbatini, A., Bensi, M., Kovačević, V., Lucchi, R. G., Morigi, C., Povea, P. and Negri, A. (2021). Benthic foraminiferal assemblages and environmental drivers along the Kveithola Trough (NW Barents Sea). Journal of Marine Systems, 224, 103616. https://doi.org/10.1016/j.jmarsys.2021.103616

## 1 Value of the Data

• This image collection represents a valuable dataset of soft-shelled foraminiferal species and morphotypes (Monothalamea, [1]) from the Arctic latitudes that provides new information regarding the abundance of this taxonomic component in this setting.
• Monothalamids are usually under-represented in traditional, morphology-based studies because of the difficulties involved morphological identification and their time-consuming nature. Such forms are often ignored when wet sediment samples are sorted for foraminifera and are not preserved when dried sediment residues are analysed, as in many geologically orientated studies.
• Monothalamids are, nevertheless, abundant and represent an important component of foraminiferal assemblages living in the marine ecosystem. The information presented will fill some potentially important gaps in knowledge that helps to manage and conserve the planet's resources responsibly.
• The photographs will be useful to scientists studying marine foraminifera by helping them to evaluate this potentially important faunal component, thereby improving understanding of foraminiferal biodiversity generally. The image dataset will be particularly valuable for micropaleontologists and biologists working on benthic foraminifera in the Arctic.

## 2 Data Description

**Fig. 1:**
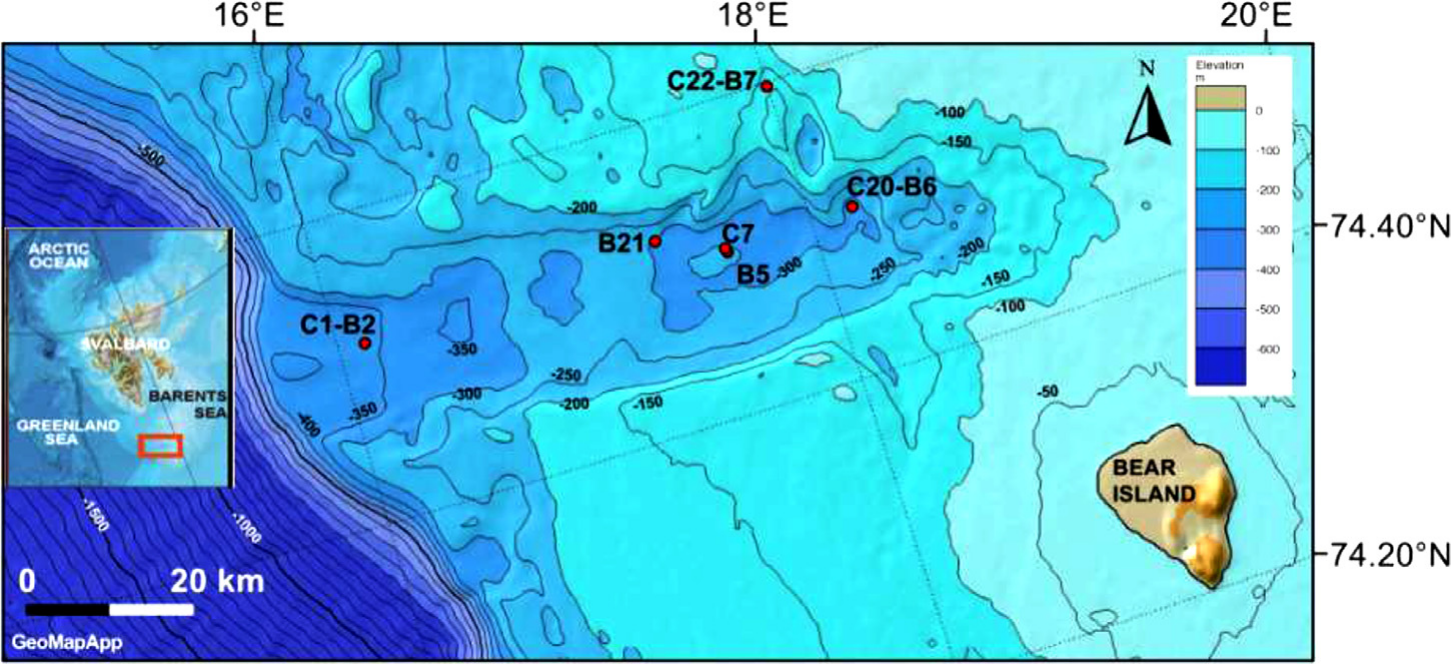
Bathymetric map of the Kveithola Trough showing the location of the cored sites (red dots) of both oceanographic expeditions. The sites with C are related to the MSM30-CORIBAR cruise while the sites with B to the Eurofleets2-BURSETER cruise.

The Kveithola Trough is an abrupt and narrow glacial sedimentary system located in the NW Barents Sea (Fig. 1). It is ca. 100 km long in an E-W direction, and less than 13 km wide with a depth range of 200–400 m along its major axis [3]. The sea floor presents a series of major, E–W trending, glacial lineationslineations related to a fast-flowing ice stream that crossed the trough during the Last Glacial Maximum, and transverse, N-S oriented Grounding-Zone Wedges (GZW) that were generated during the episodic retreat of the last glacial ice sheet during the last glacial termination. These N-S features giving rise to a stepped bathymetric axial profile of the trough [4], [5]. The inner part of the trough hosts a complex sediment drift characterized by two main depocentres (main and minor drifts; [6], with internal acoustic reflectors in the sub-bottom record indicating persistent bottom currents that were active in the area since at least 13 cal ka BP [5,6]. Furthermore, the trough is intersected in a N-S direction by the Hornsund and Knølegga fault systems, the latter being responsible for an elongated bathymetric depression designated as the “northern channel” [7,8] that conducts dense bottom currents delivering sediments towards the main drift [6,[9], [10], [11]]. Data on the morphological and structural characteristics of the sediment drift obtained during the two cruises revealed a highly dynamic depositional environment with strong bottom currents responsible for drifts formation [10]. In addition, lithofacies characteristics of surface sediments indicate, in the inner part of the trough, low-energy and/or low-oxygen conditions with black sediments containing hydrogen sulfide [6,8]. A few tens of km further west in the outer part, however, the sediments appear fully oxygenated, characterized by fine-grained, clean sands at the sediment surface with large scale ripple-like features suggesting the presence of moderately strong and persistent bottom currents.

**Plate 1:**
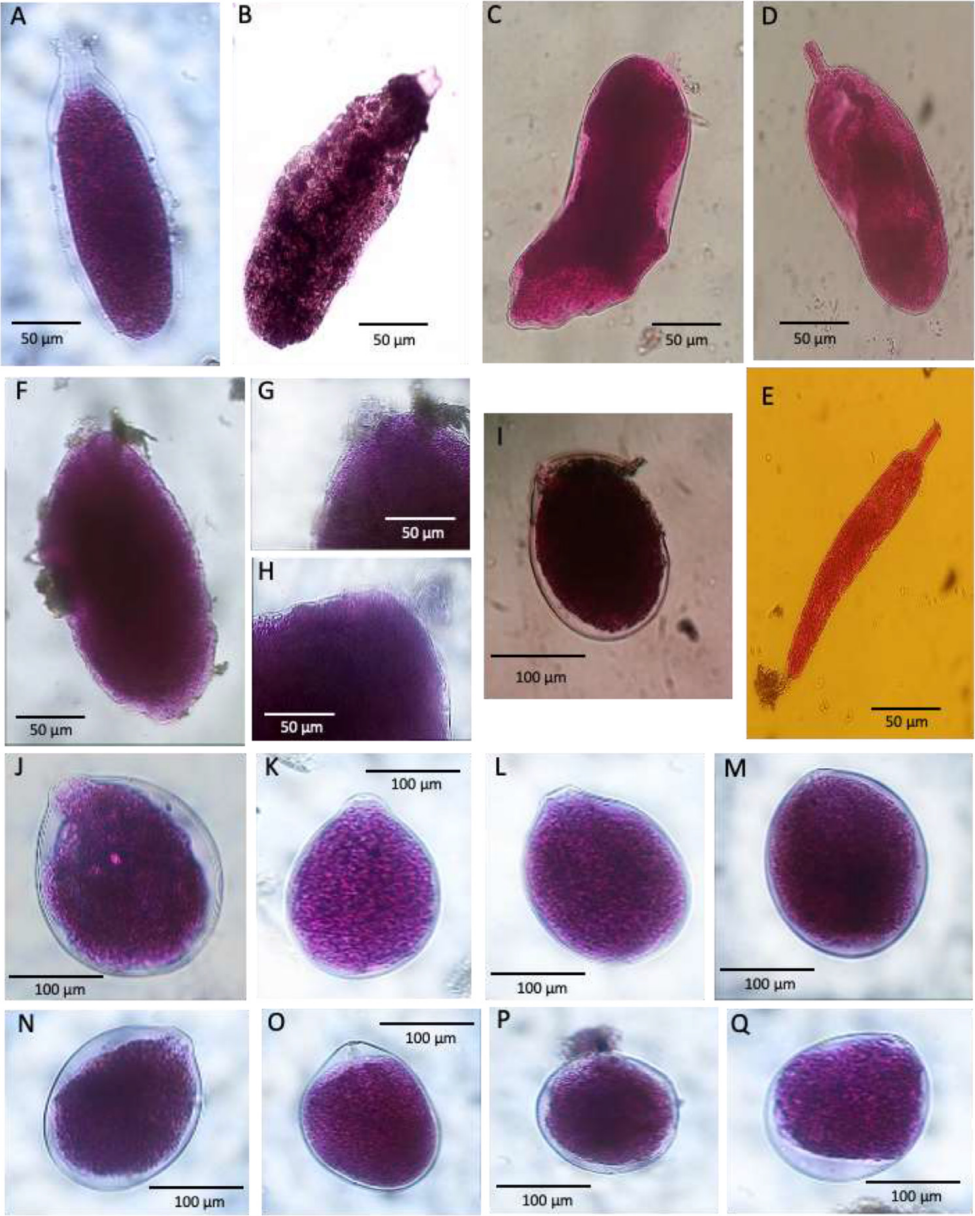
A. Allogromiid sp. B, station 01 (CORIBAR); B. Allogromiid sp. F, station 04 (BURSTER); C. Allogromiid sp. F1, station 04 (BURSTER); D. Allogromiid sp. F3 (oval test), station 04 (BURSTER); E. Allogromiid sp. F3 (elongate test), station 04 (BURSTER); F. Allogromiid sp. I, station 07 (CORIBAR); G-H. Detail of apertural region of Allogromiid sp. I (aperture), station 07 (CORIBAR); I. Allogromiid sp. G, station 04 (BURSTER); J-Q. Allogromiid sp. G, station 22 (CORIBAR).

**Plate 2:**
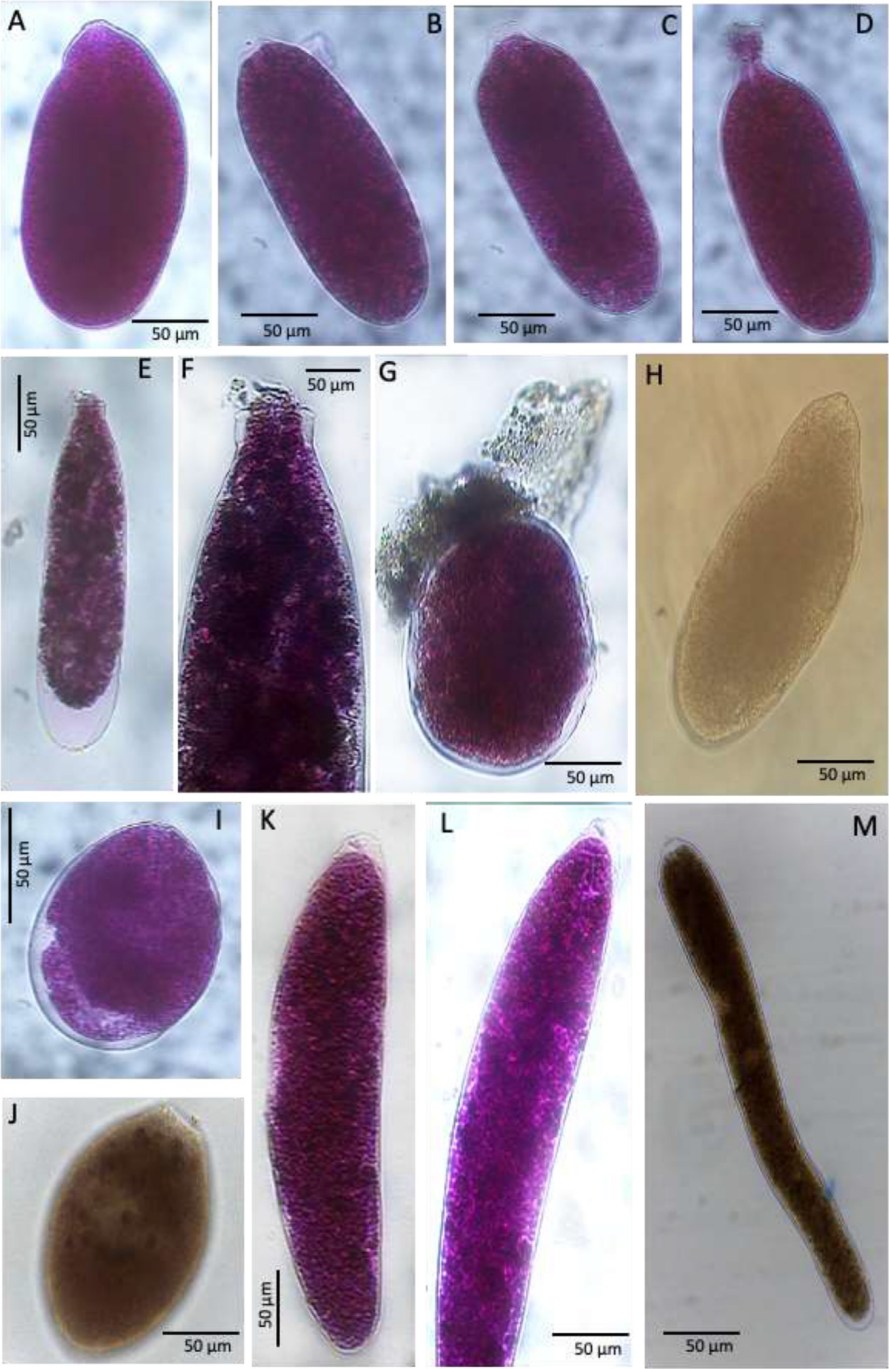
A-D. Allogromiid sp. 3, stations 01 and 07 (CORIBAR); E. Allogromiid sp. 6, station 07 (CORIBAR); F. Detail of apertural region of Allogromiid sp. 6; G. Allogromiid sp. 7, station 07 (CORIBAR); H. Allogromiid sp. 9, station 07 (BURSTER); I. Allogromiid sp. 13, station 07 (CORIBAR); J. Allogromiid sp. 35 (oval test), station 02 (BURSTER); K. Allogromiid sp. 35 (elongate test), station 07 (CORIBAR); L. Detail of cytoplasm of Allogromiid sp. 35; M. Allogromiid sp. 35 (elongate test), station 07 (BURSTER).

**Plate 3:**
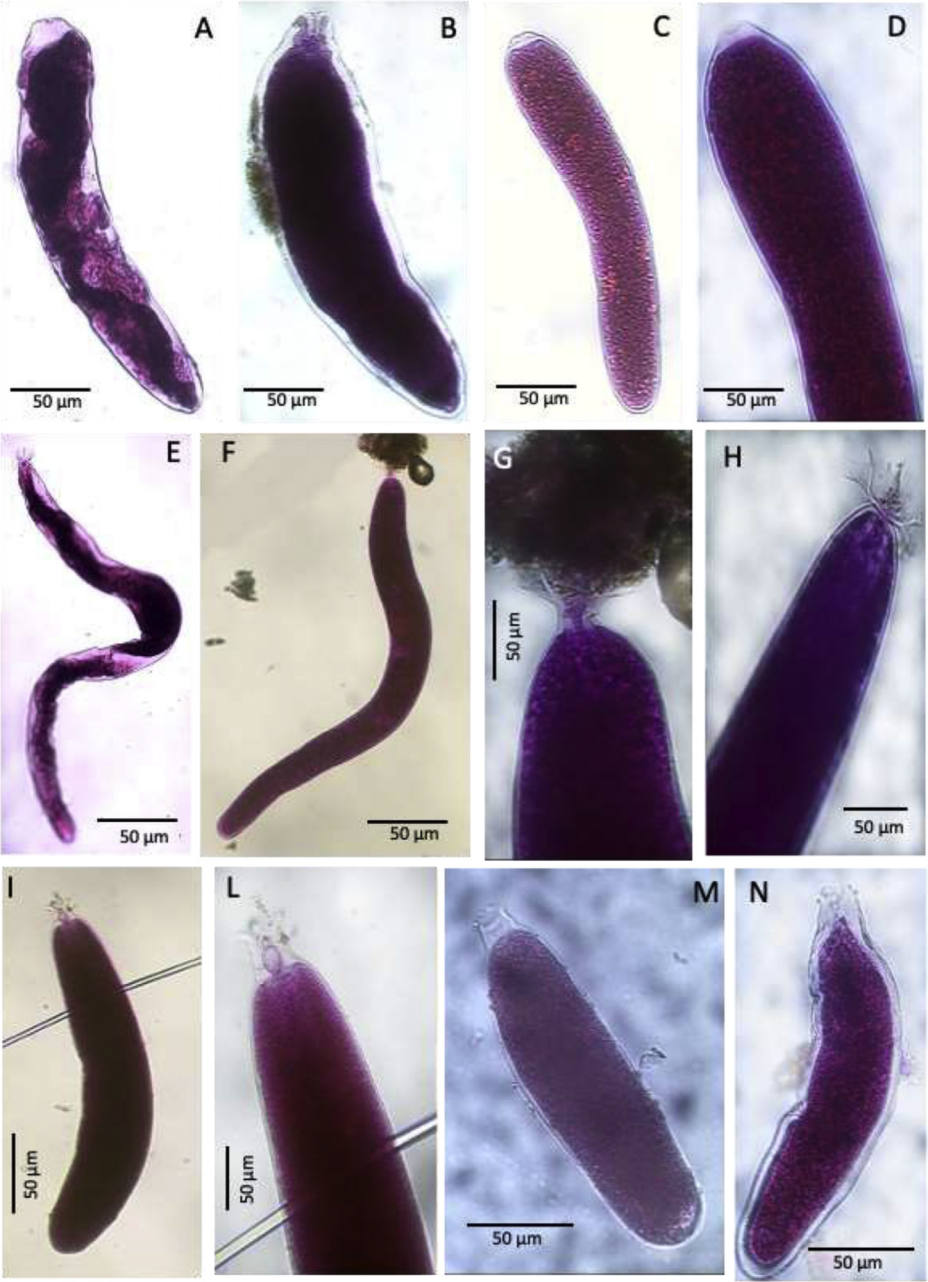
A-B. *Bowseria*-like, stations 02 (BURSTER) and 22 (CORIBAR); C. Elongate_allogromiid, stations 01 (CORIBAR); D. Deatil cytoplasm and aperture of Elongate_Allogromiid; E-F. *Cylindrogullmia*-like, station 07 (BURSTER); G-H. Details of apertural region of *Cylindrogullmia*-like; I. *Gloiogullmia*- like, stations 07 (CORIBAR); L. Detail of apertural region of *Gloiogullmia*- like; M-N. Gloiogullmia- like, stations 01 (CORIBAR).

**Plate 4:**
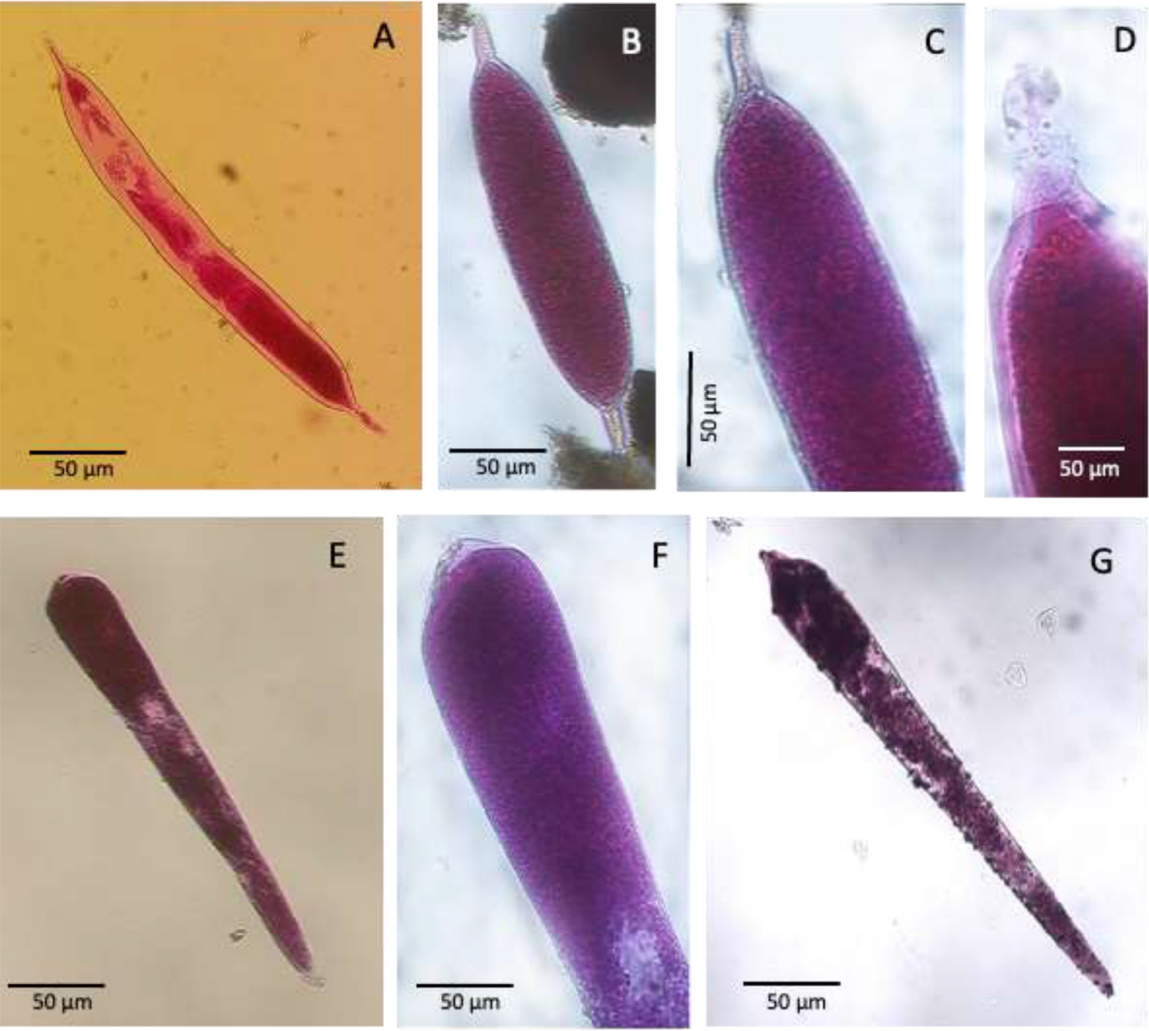
A-B. *Tinogullmia*-like, stations 07 (BURSTER-CORIBAR); C-D. Details of cytoplasm and aperture of *Tinogullmia*-like; E-G. *Micrometula* sp., station 07 (BURSTER); F. Details of apertural region of *Micrometula* sp.

**Plate 5:**
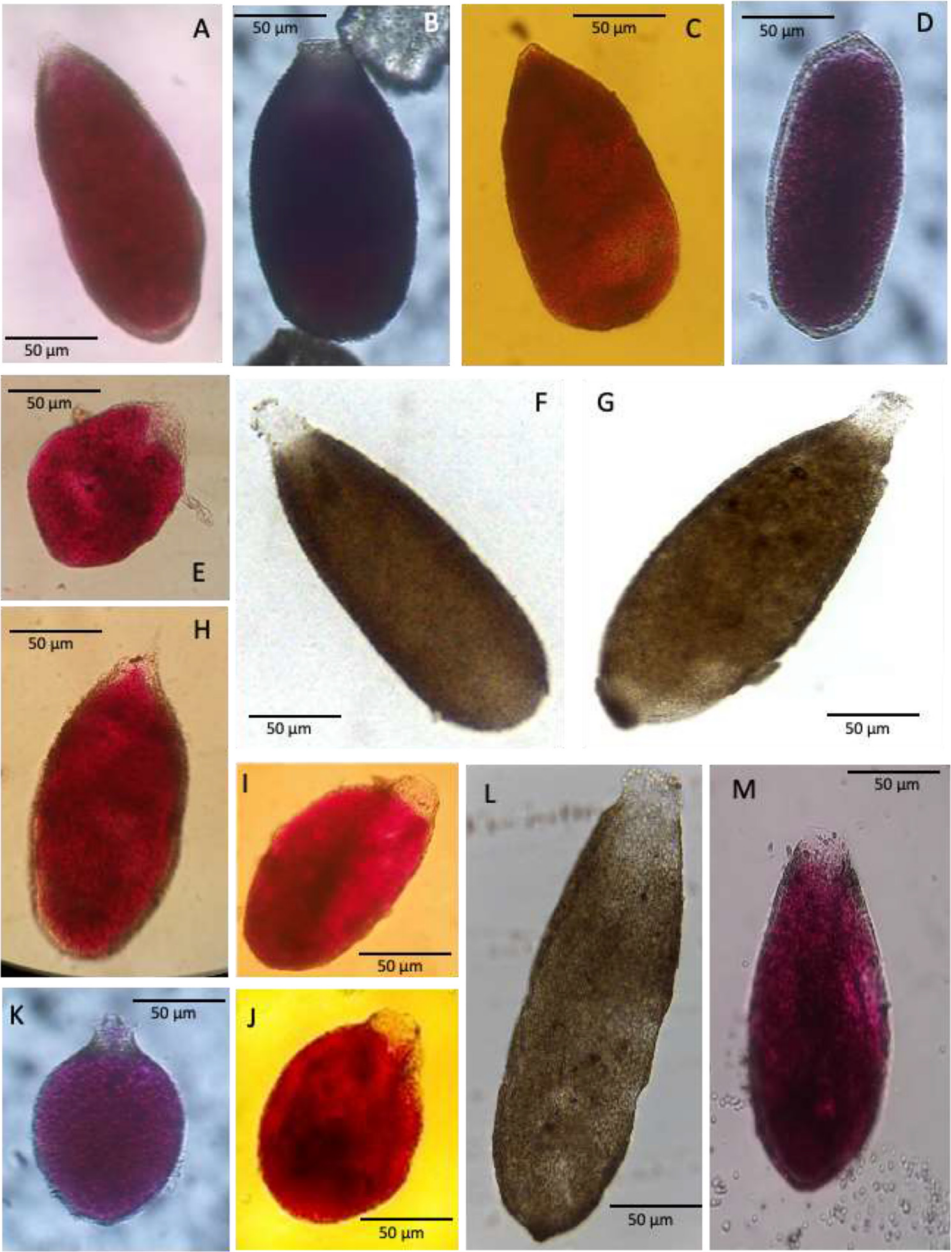
A. Saccamminid sp.1 (silver), station 07 (CORIBAR); B. Saccamminid sp.2 (white dull), station 01 (CORIBAR); C-D. Saccamminid sp.4, stations 02 and 04 (BURSTER – CORIBAR); E-G. Saccamminid sp.7, stations 04 and 07 (BURSTER); H. Saccamminid sp.10, station 05 (BURSTER); I-J. Saccamminid sp.11, station 04 (BURSTER); K. Saccamminid sp.12, station 01 (CORIBAR); L. Saccamminid sp.27, station 07 (BURSTER); M. Saccamminid sp. F2, station 04 (CORIBAR).

**Plate 6:**
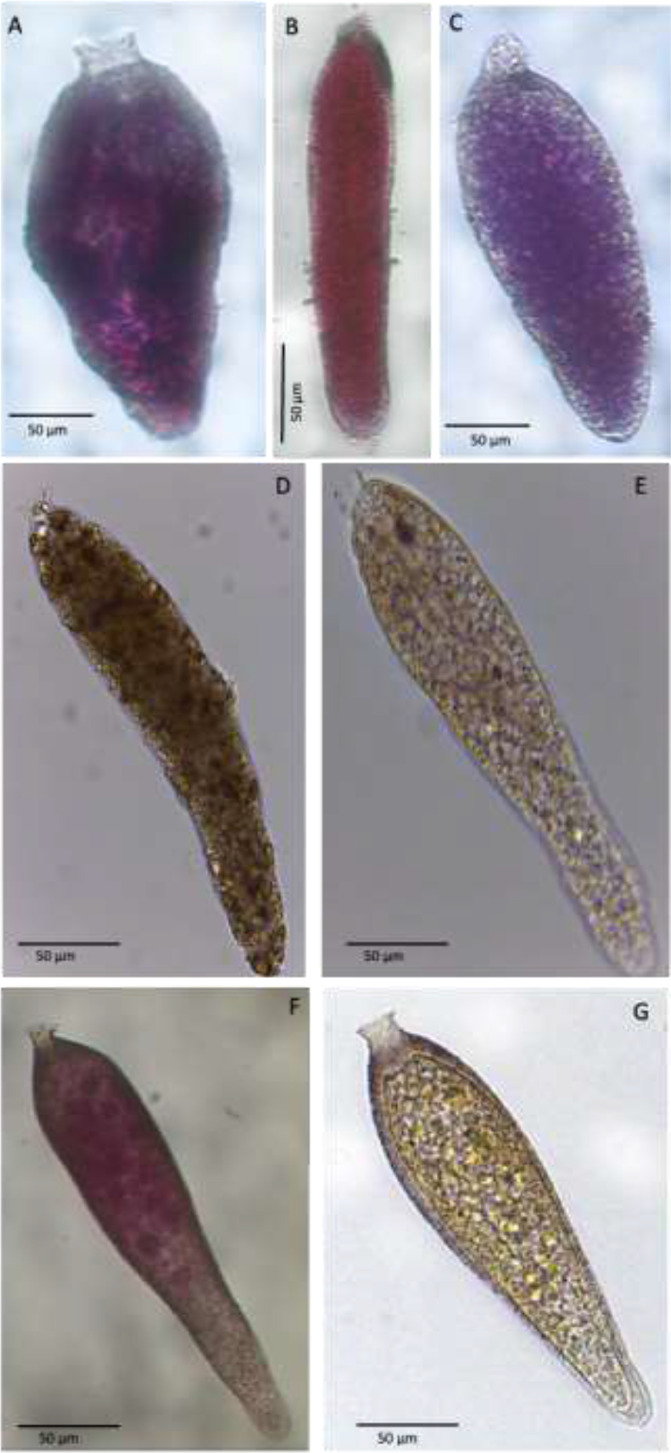
A. Saccamminid sp., station 20 (CORIBAR); B. Saccamminid sp. A/C, station 07 (CORIBAR); C. Saccamminid sp.I, station 20 (CORIBAR); D-E. Vellaria-like, station 07 (BURSTER); F-G. Conqueria-like, stations 07 (CORIBAR) and 21 (BURSTER).

**Plate 7:**
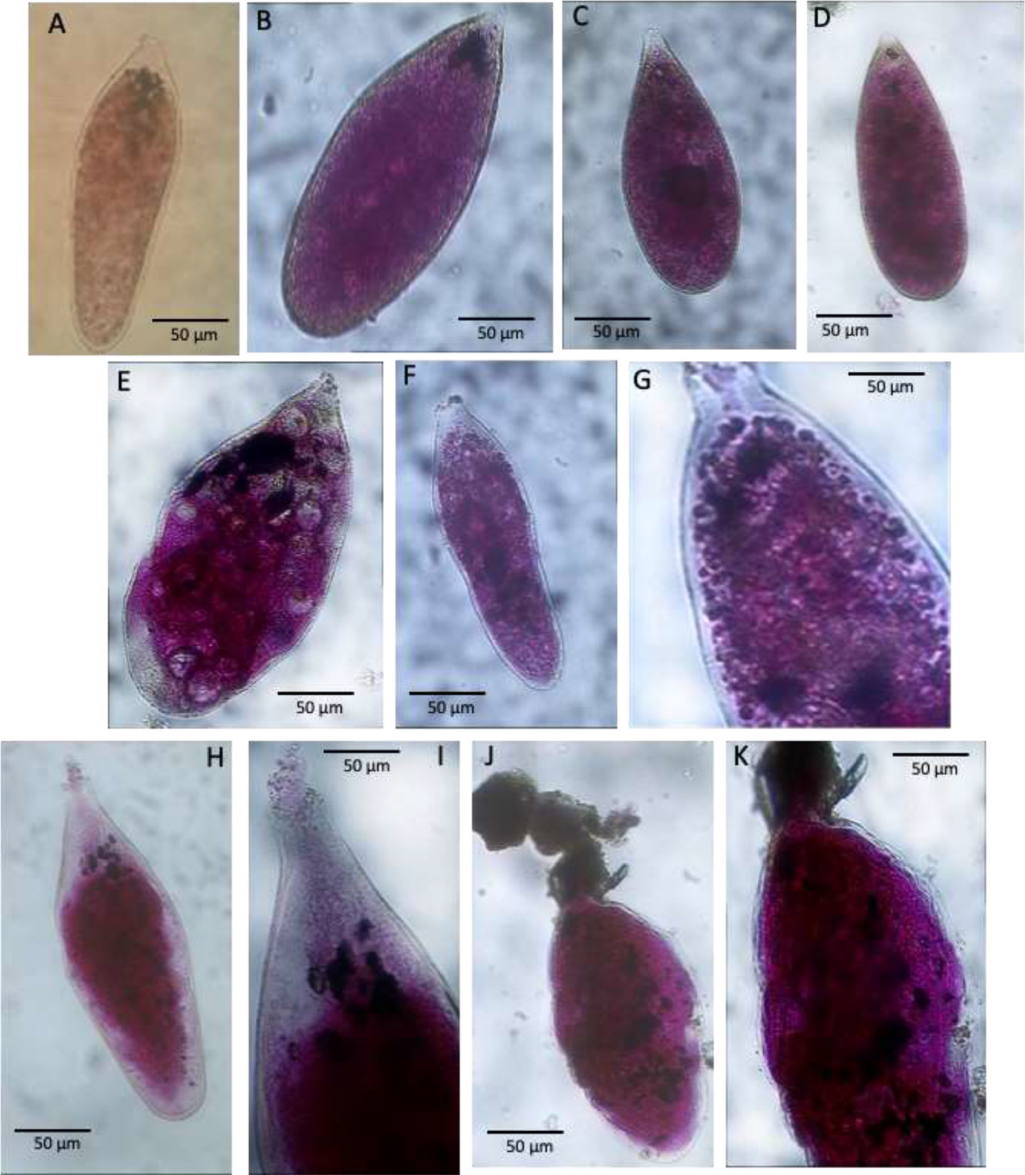
A. *Psammophaga* sp. F (arctica), station 02 (BURSTER); B. *Psammophaga magnetica*, station 07 (CORIBAR); C-F. *Psammophaga* sp., stations 02 and 20 (BURSTER-CORIBAR); G. Detail of apertural region of *Psammophaga* sp.; H. *Psammophaga* sp.1, station 07 (CORIBAR); I. Detail of apertural region of *Psammophaga* sp.1; J. *Psammophaga crystallifera*, station 01 (CORIBAR); K. Detail of cytoplasm and aperture of *Psammophaga crystallifera*.

**Plate 8:**
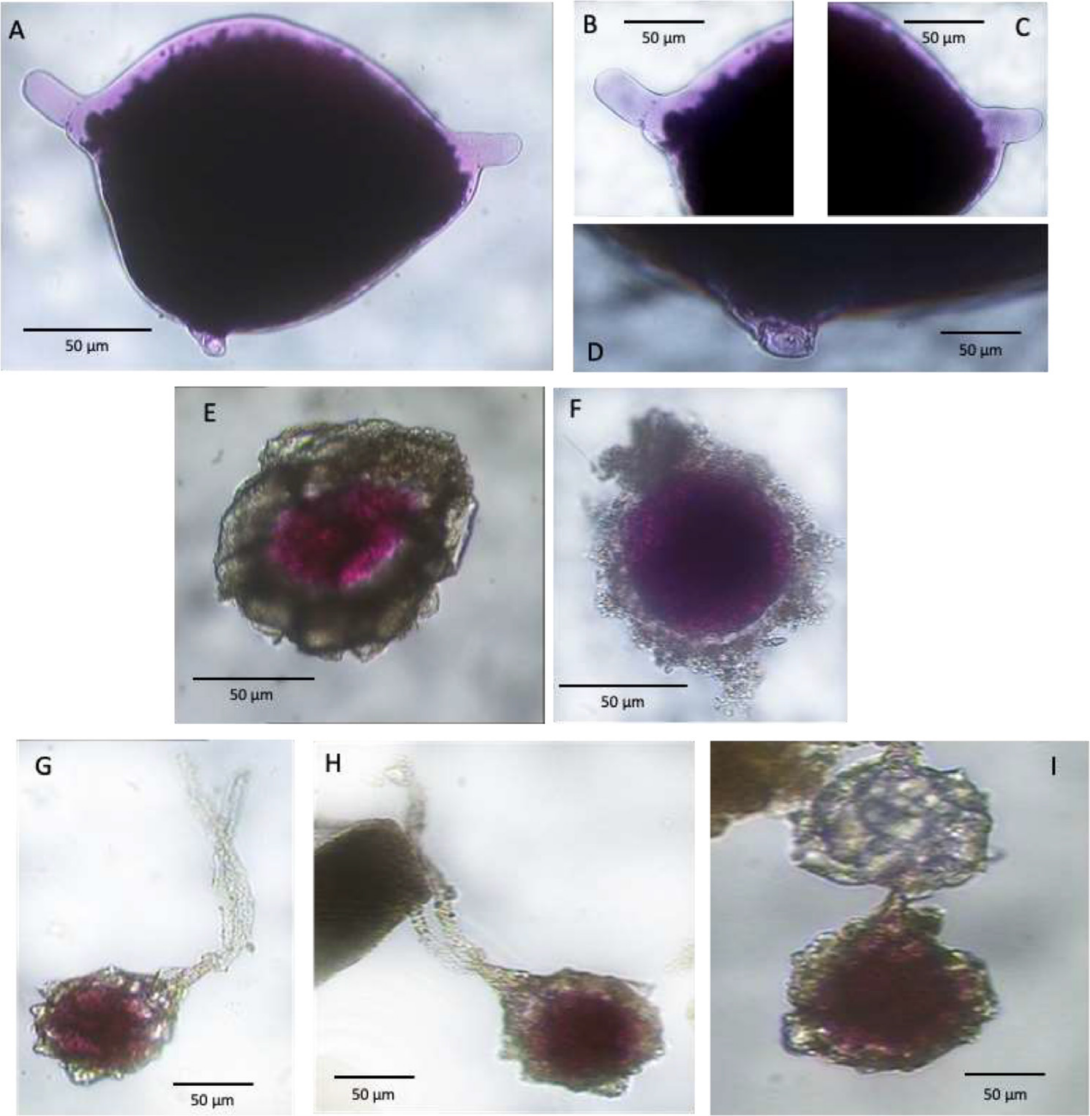
A. Thurammina-like, station 01 (CORIBAR); B. Details of apertural region of Thurammina-like; E. Psammosphaerid sp. 3, station 05 (CORIBAR); F. Psammosphaerid sp. C, station 07 (CORIBAR); G-I. Lagenammina sp., station 07 (CORIBAR).

The dataset featured in this study is uploaded on Mendeley data. The dataset is available as two folders:

- *Images_ raw data* contains 86 images of monothalamous soft-shelled foraminiferal species and morphotype.

- *Plate_secondary data* contains 8 phototables (Plates 1-8) with modified images of the monothalamous soft-shelled foraminiferal species and morphotype. Plates 1-4 contain images about individuals belonging to the family Allogromiidae characterized by organic (proteinaceous) wall, while plates 5-8 contain images about individuals with agglutinated wall belonging to the family Saccamminidae.

(DOI: 10.17632/zrynydx7py.1)

## 3 Experimental Design, Materials and Methods

The sediment dataset presented in this paper were collected during the oceanographic cruise MSM30-CORIBAR (Ice dynamics and meltwater deposits: coring in the Kveithola trough, NW Barents Sea), German R/V Maria S. Merian, 16.07–15.08.2013, Tromsø (Norway)—Tromsø (Norway) and PS99-1a, Eurofleets2-BURSETER (Bottom Currents in a Stagnant Environment), German R/V Polarstern, 13/06/2016–23/06/2016, Bremerhaven (D)—Longyearbyen (NOR)).

The sediment cores were collected along a W-E (outer-inner) transect in the Kveithola Trough, at water depths ranging from 376 m to 159 m (Fig. 1). During the MSM30-CORIBAR cruise sediment core sampling was performed using a multi-corer (MUC) equipped with eight plastic liners with a length of 50 cm and an internal diameter of 6 cm, and a giant box corer (GCB) with a 50 × 50 × 50 cm steel box. Subsamples were obtained using Plexiglas corers, internal diameter 3.6 cm, inserted manually into the GBC or MUC. During the Eurofleets2-BURSTER cruise, samples were collected using a video-guided multi-corer (TV-MUC) equipped with eight plastic liners with a length of 50 cm and an internal diameter of 6 cm that was sub-cored in the same way (Table 1).

**Table 1:** Details of core samples collected during the oceanographic cruises in the Kveithola Trough. The table includes: the cruise name, the morphology of the sea floor, the geographic coordinates, depth, sampling (MUC= multi-corer; GBC= giant box-corer) and labeled methods (RB= Rose Bengal; CTG= Cell Tracker Green) of all studied stations.

Cruise	Site	Sea Floor Morphology	Coordinates	Depth(m)	Sampling	Staining method
**MSM30-CORIBAR**	01	Grounding-Zone Wedges (GZW	74° 51.530′ N 16° 05.930′ E	376	MUC	RB
	07	Main drift	74° 50.740′ N 17° 38.350′ E	298	MUC	RB
	20	Minor drift	74° 50.740′ N 17° 38.350′ E	335	GBC	RB
	22	Northern channel	74° 59.690′ N 17° 59.590′ E	159	MUC	RB
**EUROFLEETS2-BURSTER**	02	Grounding-Zone Wedges (GZW	74° 51.530′ N 16° 05.930′ E	376	MUC	RB/CTG
	21	Main drift	74° 52.400′ N 17° 21.570′ E	306	MUC	RB/CTG
	05	Main drift	74° 51.530′ N 17° 38.370′ E	293	MUC	RB/CTG
	06	Minor drift	74° 50.750′ N 18° 10.550′ E	335	MUC	RB/CTG
	07	Northern channel	74° 59.690′ N 17° 59.720′ E	159	MUC	RB/CTG

We used two different staining methods, Cell Tracker Green (CTG) and Rose Bengal (RB), to distinguish between living and dead foraminifera. Samples collected in 2013 during the MSM30-CORIBAR cruise were stained only with RB while those collected in 2016 during the Eurofleets2-BURSETER cruise were stained using both staining methods (Table 1). CTG-labelled cores for each site were sliced horizontally onboard at 0.5 cm intervals for the uppermost 2 cm and at 1 cm intervals between 2 and 10 cm core depth, except for the core 21 where the recovery was only to 7 cm depth. Each slice was incubated in a refrigerator for 12–15 h in Cell Tracker Green CMFDA (CTG) (1 μM final concentration), following the staining procedure described [12]. After incubation, the samples were fixed in 10 % formalin buffered with sodium borate solution. RB-labelled cores from both cruises were frozen on board at −20 °C and sliced as described above at the laboratory of Paleoecology of the Department of Life and Environmental Science (DISVA_Italy). The sediment slices were then stained with Rose Bengal (1 g *L* − 1) and fixed in 4 % formalin buffered with sodium borate solution for 48 h.

After the RB and CTG staining, all sediment samples were washed and sieved through 63 and 150 μm mesh sieves to evaluate the density, size structure and taxonomic composition of the entire foraminiferal assemblage. The residues were kept wet and hand-sorted in water, all the specimens were counted, and their numbers standardized to 10 cm^2^, or if necessary 50 cm^2^, in order to compare our results with literature data. The images presented here include only monothalamous species and morphotypes (Table 2 and Plate 1, Plate 2, Plate 3, Plate 4, Plate 5, Plate 6, Plate 7, Plate 8). Specimens were placed in cavity slides in glycerol and photographed under a compound microscope (Nikon Eclipse E 600 POL) equipped with a Zeiss camera (Axiocam ERc 5 s, TV-Lens C-0.45x Nikon Japan) and fluorescence stereomicroscope Leica M205 FCA equipped with the software Leica Application Suite X (LASX).

**Table 2:** Taxonomic list of the soft-shelled foraminifera species and morphotypes picked from sediment samples.

List of Monothalamous Soft-Walled Taxa	
Allogromiid sp. B	Plate 1
Allogromiid sp. F	Plate 1
Allogromiid sp. F1	Plate 1
Allogromiid sp. F3 (oval test)	Plate 1
Allogromiid sp. F3 (elongate test)	Plate 1
Allogromiid sp. I	Plate 1
Allogromiid sp. G	Plate 1
Allogromiid sp.3	Plate 2
Allogromiid sp.6	Plate 2
Allogromiid sp.7	Plate 2
Allogromiid sp.9	Plate 2
Allogromiid sp.13	Plate 2
Allogromiid sp.35 (oval test)	Plate 2
Allogromiid sp.35 (elongate test)	Plate 2
*Bowseria*-like	Plate 3
*Cylindrogullmia*-like	Plate 3
*Conqueria*-like	Plate 6
Elongate_allogromiid	Plate 3
*Gloiogullmia-* like *Lagenammina* sp.	Plate 3 Plate 8
*Micrometula* sp.	Plate 4
*Psammophaga* sp.	Plate 7
*Psammophaga* sp.1	Plate 7
*Psammophaga* sp. F (arctica)	Plate 7
*Psammophaga crystallifera Dahlgren, 1962*	Plate 7
Psammosphaerid sp. C Psammosphaerid sp.3	Plate 8 Plate 8
Saccamminid sp. A/C Saccamminid sp. F2 Saccamminid sp. I	Plate 6 Plate 7 Plate 6
Saccamminid sp.	Plate 5
Saccamminid sp.1 (silver)	Plate 5
Saccamminid sp.2 (white dull)	Plate 5
Saccamminid sp.4	Plate 5
Saccamminid sp.7	Plate 5
Saccamminid sp.10	Plate 5
Saccamminid sp.11	Plate 5
Saccamminid sp.12	Plate 5
Saccamminid p.27	Plate 5
*Thurammina*-like	Plate 8
*Tinogullmia-*like	Plate 4
*Vellaria-like*	Plate 6

Species identification followed previous studies from the high latitude environments and in particular for the soft-shelled monothalamous foraminifera taxonomy we followed the scientific works conducted by [13], [14], [15], [16].

[2] described the population density, biodiversity and vertical distribution in the sediment samples of benthic foraminifera collected during the oceanographic cruise Eurofleets2-BURSETER, and their relationship to environmental parameters, in the Kveithola Trough. Whereas, data of the living (stained) benthic foraminiferal assemblage, collected during the oceanographic cruise MSM30-CORIBAR in the Kveithola trough, were presented by [17] with data of quantity and biochemical composition of the sedimentary organic matter.



## Limitations

None.

## Ethics statement

Hereby, I **Francesca Caridi** consciously assure that for the manuscript “Monothalamous soft-shelled foraminiferal image dataset from the Kveithola Trough (NW Barents Sea).” the following is fulfilled:

(1) All authors have been personally and actively involved in substantial work leading to the paper and will take public responsibility for its content.
(2) This material is the authors' own original work, which has not been previously published elsewhere.
(3) All sources used are properly disclosed (correct citation). Literally copying of text must be indicated as such by using quotation marks and giving proper reference.
(4) The raw data will be prepared to provide public access to such data.
(5) The paper is not currently being considered for publication elsewhere.
(6) The paper properly credits the meaningful contributions of co-authors and co-researchers.
(7) The authors declare that they have no known competing financial interests or personal relationships that could have appeared to influence the work reported in this paper.
(8) The current work does not involve human subjects, animal experiments, or any data collected from social media platforms.

## CRediT authorship contribution statement

**F. Caridi:** Investigation, Resources, Data curation, Writing – original draft. **A. Sabbatini:** Conceptualization, Investigation, Writing – review & editing, Supervision. **C. Morigi:** Conceptualization, Writing – review & editing, Funding acquisition.

## Data Availability

Soft-shelled foraminiferal image dataset (Original data) (Mendeley Data)
